# Increasing access to institutional deliveries using demand and supply side incentives: early results from a quasi-experimental study

**DOI:** 10.1186/1472-698X-11-S1-S11

**Published:** 2011-03-09

**Authors:** Elizabeth Ekirapa-Kiracho, Peter Waiswa, M Hafizur Rahman, Fred Makumbi, Noah Kiwanuka, Olico Okui, Elizeus Rutebemberwa, John Bua, Aloysius Mutebi, Gorette Nalwadda, David Serwadda, George W Pariyo, David H Peters

**Affiliations:** 1 Department of Health Policy, Planning and Management, School of Public Health, Makerere University College of Health Sciences P.O.Box 7072, Kampala, Uganda; 2 Department of International Health, Johns Hopkins University Bloomberg School of Public Health, 615 North Wolfe Street, Baltimore, MD 21205, USA; 3 Department of Nursing,School of Health Sciences, Makerere University College of Health Sciences, P.O.Box 7072, Kampala, Uganda

## Abstract

**Background:**

Geographical inaccessibility, lack of transport, and financial burdens are some of the demand side constraints to maternal health services in Uganda, while supply side problems include poor quality services related to unmotivated health workers and inadequate supplies. Most public health interventions in Uganda have addressed only selected supply side issues, and universities have focused their efforts on providing maternal services at tertiary hospitals. To demonstrate how reforms at Makerere University College of Health Sciences (MakCHS) can lead to making systemic changes that can improve maternal health services, a demand and supply side strategy was developed by working with local communities and national stakeholders.

**Methods:**

This quasi-experimental trial is conducted in two districts in Eastern Uganda. The supply side component includes health worker refresher training and additions of minimal drugs and supplies, whereas the demand side component involves vouchers given to pregnant women for motorcycle transport and the payment to service providers for antenatal, delivery, and postnatal care. The trial is ongoing, but early analysis from routine health information systems on the number of services used is presented.

**Results:**

Motorcyclists in the community organized themselves to accept vouchers in exchange for transport for antenatal care, deliveries and postnatal care, and have become actively involved in ensuring that women obtain care. Increases in antenatal, delivery, and postnatal care were demonstrated, with the number of safe deliveries in the intervention area immediately jumping from <200 deliveries/month to over 500 deliveries/month in the intervention arm. Voucher revenues have been used to obtain needed supplies to improve quality and to pay health workers, ensuring their availability at a time when workloads are increasing.

**Conclusions:**

Transport and service vouchers appear to be a viable strategy for rapidly increasing maternal care. MakCHS can design strategies together with stakeholders using a learning-by-doing approach to take advantage of community resources.

## Background

The fifth Millennium Development Goal (MDG) is to reduce maternal mortality by seventy five percent. The demographic health surveys done in Uganda indicate that maternal mortality has been persistently high (527 in 1995, 505 in 2000, and 435 in 2005) [1]. There is evidence to show that attendance at delivery by skilled health personnel reduces maternal mortality [2], but over one half of all women in developing countries do not have a skilled birth attendant at delivery [3]. In Uganda the proportion of mothers delivering in health facilities has been persistently low (38% in 1995, 37% in 2001 and 41% in 2006), but the rates are even lower in rural areas and among the poorest quintile of the population [1].

In Uganda, studies have shown that the main reasons for women not delivering in a health facility include: overall financial limitations, long distances to health facilities coupled with transport difficulties, lack of decision making power among women, inability to afford the medical supplies that are often compulsory at public health facilities, rude health workers, and preference for traditional child birth positions [4-7]. As in many other countries, maternal health services have typically been provided by both the public sector and the private sector. The public sector in Uganda has several shortfalls which include a shortage of health workers, inadequate supplies, unofficial charges, and unsympathetic health workers [8,9]. Financing of health services is mainly through tax based public subsidies’ although the literature shows that the poor often do not benefit from public subsidies. Some of the reasons given include distance from facilities, leakage of resources away from the diseases common among the poor, ignorance of treatment options, and cultural and household constraints [10].

Ensuring access to quality maternal health care services throughout pregnancy and childbirth is therefore essential. Lack of proper provision of essential obstetric care services has potential life threatening effects, and may lead to serious complications which require expensive specialized treatment that is extremely costly relative to disposable income [11]. However, access to such services for the poor is often limited [12]. Some attempts have been taken to increase institutional deliveries from the supply side. These include training of midwives in safe motherhood and life saving skills; construction of level IV Health Centres that are expected to offer emergency obstetric care; and training of comprehensive nurses who can offer both midwifery and nursing care. However, there have been no concerted efforts to intervene on the demand side.

Financing of maternal health services is also largely supply based, is not directly linked to the quantity or quality of services, and does not consider the provider’s ability to reach vulnerable populations. The nature of maternal health services makes it imperative that financially sustainable strategies are put in place to ensure access. Financing of health care may involve demand or supply side approaches. When supply side approaches are used, resources are allocated to a supplier so that they can provide particular services based on the cost of inputs. Utilizing the demand side approach, financing is made through patients, giving them the purchasing power so that providers are then paid based on the quantity and/or quality of services they provide [13]. A combined demand and supply side based system of financing, where facilities are partly funded based upon their ability to provide services to targeted populations, may result in improved benefits for the poor and vulnerable.

Innovations in financing such as conditional cash transfers, vouchers, provider subsidies and equity funds may have the potential to increase access to health services by the poor [12,14-16]. Demand side approaches often utilise vouchers. Consumers are given a written entitlement which can be exchanged for a specified service up to a pre-determined amount at accredited facilities. Alternatively, consumers are told to claim a given service from a provider who then claims payment directly from the financing agency [13]. Pearson defined such consumer led demand side financing mechanisms as a “transfer of purchasing power to specified groups for defined goods and services” [17]. Experience suggests that demand side financing using vouchers has a potential to provide more targeted services to the poor [13]. A study in Nicaragua showed that vouchers could substantially increase the use of services for sexually transmitted infections, demonstrating that many adolescents were willing to use such services if readily accessible [18]. In Tanzania the use of vouchers increased the use of insecticide treated nets (ITN) among pregnant women [19]. In Bangladesh they reported increased deliveries attended by skilled birth attendants among the poor [20]. Furthermore, it has been suggested that competitive voucher schemes are able to target subsidies more accurately, provoke demand for under used services, and may lead to improvements in technical quality [21].

Although consumer led demand side financing can be used in a number of different ways to further public policy objectives, it is important to note that its implementation requires a concerted effort to address various pitfalls. Others have noted that such schemes may lead to over servicing where providers provide more services than necessary because of the link between the outputs and the finances earned. These schemes are also prone to cream skimming where providers choose to offer services to clients who have fewer problems. In addition the transaction and administration costs for implementing the scheme can be particularly high [21]. Geographic accessibility of services and a multiplicity of providers are also essential to ensure that the target group obtains a desired service [13].

Mechanisms for increasing awareness about the scheme must also be put in place. In the Tanzania study the voucher return rate was extremely high at 97% (7720/8000). However, two years after the start of the program awareness among target groups was only 43% [19]. Additional pre-requisites for success included a strong administrative capacity for implementation, an accreditation system or at least a system of ensuring quality of care in health facilities involved in the program, and a methodology and capacity for identifying target groups [13,22]. In summary, the available literature shows that voucher schemes are a potentially effective means of targeting health services to specific population groups such as pregnant women, children under five, or the poorest. However, most voucher schemes to date have been implemented on a small scale. There is limited documented evidence of their successes or of the feasibility and cost implications for scaling up pilots [13,22].

MakCHS is undergoing a transformation with the aim of playing a stronger role in improving health outcomes in Uganda. We present here a study that was designed to demonstrate how ongoing reforms of the College can lead to new ways in which the College can work with local stakeholders, to influence the health system, with the ultimate aim of improving health status in Uganda. At the time of writing, the study is still in progress, but early results on the use of services are available from the routine information systems and are presented.

The intention of the study is to assess the effectiveness of a specific demand and supply side financing system using vouchers to increase use of maternal health services at health units. The information obtained will be used to guide decisions that are geared at promoting the attainment of the 5^th^ MDG. It is envisioned that the availability of increased research evidence in this area will encourage greater use of cost-effective lifesaving interventions. The primary research questions for the study are: Does providing a financial incentive (a voucher for institutional delivery and a voucher for transport to access antenatal, delivery and postnatal services) increase antenatal, institutional delivery, and postnatal attendance, using public, private-not-for-profit (PNFP), and private-for-profit (PFP) health facilities?

Our hypotheses are that women in areas where the intervention is provided are more likely to deliver in health facilities, attend more antenatal and postnatal care compared to women in areas where no intervention is offered. Broadly, the study is designed to

1. Identify the demand side factors that influence delivery at health facilities.

2. Evaluate the effectiveness of a service and transport voucher system in increasing deliveries at public, PNFP and PFP health facilities, and among the poor in particular.

3. Identify the pathways through which vouchers influence delivery care services at public, PNFP and PFP health facilities.

4. Estimate the incremental cost of implementing a voucher system to increase deliveries at health facilities.

Since the study is ongoing at the time of writing, in this article we provide detailed information on the study protocol, and report on the early results on the use of antenatal, delivery, and post-natal services. We also discuss the lessons learned by the implementers in how to design and implement this type of strategy that involves closely working with rural communities.

## Methods

### Study design and setting

The study is being implemented in four health sub-districts (HSD) within the two districts of Kamuli and Pallisa. A HSD is a region in the district with geographical and administrative boundaries, with an estimated population of about 100,000 people, 8-12 lower level health units, and is headed by a medical doctor. Each HSD headquarters has a surgical theatre for emergency obstetric surgery – but most are not operational due to resource and management constraints. Each of the two districts where the study is being implemented has one HSD in the intervention and one HSD in the control arm. The initial pilot study for which these results are reported was done in Kamuli district.

### Study population

In the intervention arm, the teams developed a program with the local population to share the intervention (voucher program). All pregnant women, irrespective of the duration of pregnancy, who are resident in the study area during the intervention program period are be enrolled into the intervention. A resident is be defined as a woman who has stayed in the study area for at least 3 months, and who intends to continue residing in the area. In the control arm vouchers are not distributed.

### Service package

The strategy has two main components: a demand side component that uses vouchers, and a health system strengthening component. The demand side component comprises of a voucher given to pregnant women for health services and a voucher for transport. The voucher for services entitles a pregnant woman to deliver in a specified health facility located within the study area regardless of the ownership. These include public, PNFP and PFP facilities. The voucher for transport services entitles the pregnant woman to use locally available transport (motorcycle, bicycle, public taxi) to the health facility for antenatal care sessions (four, three, two or one antenatal care session depending on the gestation of pregnancy), one delivery care session, and one post natal care session. Women who are referred from a lower level facility to a higher level facility for a caesarean section receive a special voucher for transport that allows them to use a motorised vehicle (public taxi or ambulance). The vouchers for maternal services are used for antenatal, delivery, and postnatal care services offered by both private and public facilities.

Modest improvements were made in the inputs to quality of care in health facilities in both the control and the intervention arms. This involved refresher training of providers on maternity care, and small supplements in essential drugs and basic supplies to assure they were available in both arms at the beginning. The intention was to improve provider capacity in both the intervention and control arms.

The intervention package described above was piloted, and based on a review of the results a decision was made to amend the protocol by redesigning the benefit package. The pilot study led to an overwhelming response, with mothers arriving in large numbers at the facilities, resulting in projected costs which were beyond the resources available to the project. In the redesigned package, the vouchers now exclude ANC but cover only the delivery care session (for all pregnant women) and one post natal care session (for a high-risk mother or a newborn with danger signs). A high risk mother is defined as a mother who had danger signs during her pregnancy, at delivery, or in the postnatal period. These two services were selected because they are considered most critical to maternal and neonatal survival, and because there were very low levels of utilization prior to the study. If a mother has a life-threatening complication during delivery or in the postnatal period, it could result in her death without immediate appropriate care. However, all mothers were still strongly encouraged to attend four antenatal and one postnatal session at the clinic.

The voucher for services during the pilot period entitled a pregnant woman to receive ANC services (4,3,2 or 1 depending on her gestation period), 1 postnatal care session (PNC) and delivery care services (including caesarean sections) in participating health facilities located within the study intervention area regardless of the ownership. These include public, private not-for-profit (PNFP) and private for-profit (PFP) facilities. The voucher for transport services during the pilot period entitled the pregnant woman to use locally available transport (motorcycle, bicycle, public taxi) to the health facility for four antenatal care sessions, one delivery care session and one post natal care session (return trip). Women who are referred from a lower level facility to a higher level facility for a caesarean section receive a special voucher for transport that allows them to use a motorised vehicle (public taxi or ambulance). The vouchers are distributed during antenatal care (ANC). Attendance of at least one ANC session in Uganda is high (94%), so it is expected that this will allow contact with the eligible respondents.

### Redemption of the vouchers

The vouchers for services are given to pregnant women who attend antenatal care and they are redeemed by the private and public facilities for cash in direct proportion to the number of deliveries, ANC and PNC sessions conducted on a monthly basis. Payment per service differs in public and private facilities since they receive different amounts of funding from the government for the provision of services. Based on the current charges for deliveries, ANC and PNC in the private facilities in the intervention area a price was set for the service voucher. The price of the voucher in the public facility was then set at 75% of these charges.

The vouchers for transport are given to pregnant women who attend antenatal care and they are redeemed by the transport providers for cash in direct proportion to the number of women whom they transported. Based on the current estimates for transport fares, the price for the transport voucher was set at 5000 Ug Sh (2.5 USD).

### Data collection

Data collection was ongoing at the time of this article. We report here the full data collection methods as per protocol, but then report on the preliminary findings from data collected at health facilities through the health management information system on the utilization of maternal services.

The data collection for the full evaluation includes baseline and endline household surveys. For the baseline household survey a sample of women were enrolled into the survey according to pre- specified eligibility criteria. The same criteria will be used for the end line survey. The inclusion criteria consisted of women who delivered babies in the 12 months preceding the survey irrespective of whether the delivery was preterm or term and the viability of the birth outcome. It included next of kin of women who died during child birth in the 12 months preceding the survey. The women had to be residents in the study area, capable of giving informed consent, and if under 18 years married and an emancipated minor who could provide individual consent. Women were excluded from the study if they did not give consent to participate, were not resident in the study area at the time of the birth of their baby, or gave birth more than one year prior to the baseline survey. For the endline survey women who gave birth prior to the start of the intervention will also be excluded.

### Sampling procedure

For the baseline assessment, multistage sampling was used to select 734 women from each HSD as detailed below.

1. The four districts in the Eastern region that had at least 3 HSDs were selected. This was to allow for the inclusion of a buffer zone between the intervention and control HSDs.

2. Two districts were selected purposively. The selection was guided by the distribution of public and private facilities, as well as the distribution of higher level facilities and lower level facilities, and the capacity to provide maternal health services.

3. All the HSDs within the two districts were then listed and the following variables were measured: level of health facilities; type and number of staff available in the health facilities; type of health facilities (public, PNFP, PFP), antenatal care coverage, institutional delivery coverage, and capacity to provide delivery care (in terms of human resources, equipment, supplies and infrastructure).

4. The HSDs which fell within a given range (excluding extreme cases) were selected as the eligible sub-districts.

5. The eligible HSDs were stratified by district and then the two HSDs which were most comparable with regard to criteria three above were selected. Each district had only three HSDs. In each district one HSD was then randomly selected as an intervention HSD using the ballot method. All the sub counties and parishes in the HSDs were then listed and 50% of them were randomly selected using the ballot method with replacement.

6. Fifteen villages were initially randomly chosen from the parishes using a table of random numbers, and a household enumeration census was conducted in these villages to identify households with eligible women. All households that had women who delivered babies in the previous one year, irrespective of whether the birth was term or not, whether the child was alive or not and, whether the mother of the baby survived the birth or not were eligible. Using this household registration system (HRS), a list of households with eligible women was made. All the eligible women were enrolled into the study.

### The study components

The study has five main phases: design phase, pilot phase, implementation phase, evaluation phase, and a dissemination phase. Table 1 summarises the activities that take place in each phase.

**Table 1:** The study phases

PHASE	ACTIVITY
Design Phase	Writing of the study proposal and seeking ethical approval
	Sensitization of leaders in the study area
	Needs assessment of the health facilities in the study area
	Survey of transport providers
	Exploratory research
	Preparation of the intervention material: manuals, vouchers
	Baseline household survey
	Training of health workers
	Preparation of facilities

Pilot Intervention Phase	Piloting of the intervention in one HSD
	Review of pilot intervention results

Intervention Phase	Distribution of vouchers to all pregnant women reporting to the facility
	Field supervision, and support supervision performance
	Audit of complications, maternal and neonatal deaths

Post Intervention Phase	Collection of additional data required for the evaluation of the effectiveness of the intervention: Household survey, focus group discussions, key informant (KI) interviews
	Estimate the cost of the intervention

Dissemination Phase	Data entry and data analysis
	Report writing
	Dissemination

### Design phase

#### Sensitization of leaders in study areas

The district leaders in the study areas were visited, informed about the study, and permission to conduct the study in the districts was obtained. Collaboration with respective health departments was deemed to be essential for the success of the study. A stakeholder’s consultation workshop was held with the district leaders during the design phase of the study so that suggestions from the district could be considered. These stakeholders included the district technical and political leaders, local district leaders, and the health workers from health units in the study area. After the piloting of the study, meetings were again held with the district level stakeholders. The study team intends to continue these meetings with the stakeholders every three months until the study is completed.

National level stakeholders were also consulted, mainly through one-on-one meetings. The team found these stakeholder consultations useful because they allowed the team to address the concerns of the stakeholders mainly related to the quality of care received by the mothers, implementation problems encountered, and the feasibility of scaling up the study.

#### Needs assessment of the health facilities in the study area

A needs assessment was done of all the facilities located within the study health sub-districts to establish their capacity to provide maternal health services. It was important that facilities participating in the study were able to provide an acceptable level of care. The assessment focused on the availability of essential drugs for delivery (ergometrine, pitocin, gentamicin, crystalline penicillin), basic equipment for delivery (ambu bags, delivery kits, mama kits, gloves, cord ligatures, mackintosh, caesarean section kits), availability of communication equipment for referral (transport, mobile phones or fixed phones, radios), and skills of staff in detection of mothers at risk, conducting deliveries and detecting complications.

#### Survey of transport providers

A mapping of the local transport providers who are available within the study area was also done. Specifically the study collected information on the type of transport available [special hire vehicles (taxi in the western world), public taxi (mini bus), motorcycle, bicycle] and amount of money charged for specified distances. This information was useful for designing the transport component. During the course of the study, the transporters started playing a very influential role - mobilising mothers to attend services and ensuring that they were transported to the facilities. It was noted that the success of the pilot was largely linked to the transport component [23]. It therefore became necessary for the team to collect sufficient monitoring and evaluation data about the transport component. The study protocol was thus amended to allow the collection of information about the socioeconomic and demographic particulars of the transporters, ownership and use of the transport vehicles, and recurrent and fixed costs of maintaining the vehicles.

#### Exploratory research

Exploratory research was conducted through focus group discussions with women of reproductive age and transport providers to collect information that would be useful for the designing of the intervention. The main objective of the focus groups with women were to determine the factors that affected delivery in the study area, the suggestions that the community had about how these factors could be addressed, and their perceptions about the proposed intervention. Six focus group discussions were done with groups of 6-10 women. Another nine focus groups were done with transport providers in the area. The main objective of the transport providers’ discussions was to obtain their views about the proposed transport scheme, their preferred modes of payment (cash or cheque) and their preferred frequency of payment.

#### Designing of service and transport vouchers

The vouchers were designed with unique features that would make it difficult to reproduce. Each voucher has a unique registration number; this will help to avoid duplication of the vouchers. The vouchers are time limited and not reusable. They have a provision for the date, name of the client, name of the issuing health facility, place of residence (parish and village), and the expected date of delivery. Other details such as the woman’s age, parity, next of kin (this information will be useful to track patients if necessary), and last normal menstrual period were recorded in the facility register. The original plan was to include such details on the voucher, however it was necessary to limit the information required to be completed since the midwives, who were completing the vouchers, were already overloaded with work. One of the risks in the study is the sale of vouchers. This behaviour was minimised by matching the personal records given by the patient at antenatal care delivery and postnatal with the information received at enrolment. Furthermore patient records from the service vouchers were also matched with the records from the transport vouchers. If improper use of vouchers is noted to be a significant problem then additional measures will be taken to minimise it.

#### Knowledge updates training of health workers, study participants

Knowledge update training of midwives in emergency obstetric care and safe motherhood was done in all facilities serving both the intervention and the control areas. Consultants with experience in conducting safe motherhood and emergency obstetric care conducted the training. This training is being reinforced throughout the study period through support supervision.

#### Preparation of facilities

The facilities that are participating in the study were equipped with the necessary materials before the piloting of the study began (Vouchers, data collection forms, registers).

### Piloting the strategy

The study was piloted in one of the intervention health sub districts. It was decided that the pilot should be done within the study area rather than a separate area because of the nature of the study. All the women enrolled into the study would continue to receive support from the study until after their postnatal period. Service and transport vouchers were given out to pregnant women. This was done for a period of twelve weeks from December 2009 to March 2010. The vouchers used during the piloting of the study had a different series of numbers from the ones used in the implementation phase. All the information recorded on the vouchers was also recorded in a register in the health facility. Additional information such as antenatal care attendance, intermittent preventive treatment for malaria (IPT) uptake, tetanus toxoid uptake, date of delivery, type of delivery (normal or caesarean section), birth outcome (live birth, still birth) and the condition of the mother was captured through the facility register. If the patient was referred to another health facility this was also recorded, as well as the reasons for the referral. The pilot provided an opportunity for the study team to detect short falls that needed to be dealt with during the study implementation. In this case it led to the redesigning of the benefit package since the original benefit package was deemed to be too expensive and was therefore likely to not be sustainable.

### Implementation of the study

#### The distribution of vouchers to pregnant mothers in all participating facilities

The vouchers were given out during the antenatal care sessions. Awareness about the voucher scheme was increased using sensitisation meetings, posters and local radio talk shows. The midwife on duty informed the mothers about the study and obtained informed consent from those who were willing to participate. Those who consented were given vouchers. Vouchers were given out for a period of three months during the pilot. During the implementation phase vouchers were to be given out to 30,000 mothers irrespective of their gestation age. This figure was determined according to the available financial resources, since the study would be obliged to meet the costs for all the mothers enrolled into the study.

#### Data collection

The midwives were responsible for collecting all the data required for the study in the intervention and control areas. A special data collection register was designed for capturing data about the clients in the facilities. However this resulted in a huge workload for the staff at the facility, due to duplication of work, and a decision was made to use the regular facility registers for data collection instead.

#### Audits of complications, maternal and neonatal deaths

Audits of complications, maternal and neonatal deaths were done at hospital level to review the management of the patients and ascertain the circumstances surrounding the deaths. Most of the maternal deaths occur either in the community or at the hospital level and not at the district level.

#### Field supervision

During the first month of the research, the members of the research team were available in the study area and control area full time to ensure that the study was progressing as planned. Thereafter a member of the research team visited the control and intervention areas every two weeks. The facilities were, however in direct telephone contact with the research team, so that any pending issues could be handled immediately.

The main purpose of the field supervision visits were to ensure that the field procedures such as the enrolment of mothers, distribution of vouchers, recording of data, and transportation of mothers were being implemented as planned. Support supervision was also done in both the control and intervention districts every six weeks by a team of midwives and obstetricians and members from the district health team. The supervision is done using support supervision guidelines from the ministry of health. The aim of the supervision visits is to encourage quality improvements by ensuring that standards for service delivery are observed and the required resources in terms of essential drugs, staff, and equipment are available.

#### Redemption of the service vouchers

The vouchers for services were given to pregnant women who attended antenatal care and the vouchers were redeemed by the private and public facilities for cash reimbursements in direct proportion to the number of deliveries, and the antenatal and postnatal sessions conducted on a monthly basis. Based on the current charges for deliveries, antenatal care and postnatal care in the private facilities in the intervention area, a price was set for the service voucher.

Most facilities were charging between 5,000 and 10,000 Uganda shillings (US$2.50 to US$5). In view of the fact that the price charged was not equivalent to the cost of providing the services, the study team decided to set the price for the delivery voucher at 15,000 Uganda shillings (US$7.50), a first antenatal visit was 2,500 and a fourth antenatal visit and postnatal visit was priced at 3,000 Uganda shillings. The price of the vouchers in the public facilities was then set at 75% of the stipulated prices. Although public facilities receive funds for service delivery from the government, these funds are usually inadequate. It was felt that the project needed to supplement what they receive, and 75% of the amount given to the private facilities was considered adequate for this purpose.

Based on the current estimates for transport fares, the price for the transport voucher was set at a flat fee of 5,000 Uganda shillings (US$2.50). This was a flat rate that didn’t vary with the distance travelled or the time of travel. Transport fares are usually higher for longer distances and at night. A flat figure was chosen because it was envisaged that it would be difficult to measure distance and confirm the time the mother was transported so 5,000 Uganda shillings (US$2.50) was considered sufficient for short and long distance journeys made both during the day and the night. However, following the pilot results these rates were revised making them dependant on the distance between the facility and the village where the transporter operates. The rate for transport to the referral district hospitals was higher than 5,000 Ugandan shillings (US$2.50) and dependent on the distance between the facility and the referral hospital.

### Monitoring and evaluation

The collection of data about the primary outcomes and secondary outcomes commenced as soon as the implementation phase started, and it will be concluded at the end of the implementation phase. The data collection methods included household surveys, focus group discussions, in-depth interviews, and surveys with the transport providers. The purpose of these qualitative and quantitative interviews was to collect the views of the clients, transporters and providers about the voucher scheme and the influence on them. During the formative and summative evaluation, it is important to evaluate the transport and service delivery component as well as to get perspectives from the community. This will help the study identify challenges encountered and means of addressing these challenges. It will also provide an opportunity to collect information that may be important for guiding decisions related to sustainability and scale up. Key informant interviews and the focus groups are being done throughout the study (every four months from the beginning of the implementation phase). Eight focus group discussions will be conducted with women of reproductive age in the study area. Two focus group discussions will be conducted in each health sub district. Six focus group discussions will also be done with transporters. Eight in-depth interviews will be done in the study area. Four will be done with health workers and four with female community leaders in the four health sub districts. Quality of care assessments will also be done at the beginning and at the end of the intervention. Data on the total number of deliveries, types of deliveries, and complications will also be collected from facility registers, which comprises the routine health information system. These data are collected every month throughout the study period. In this paper, we report on utilization of maternal services based on the health information system.

### Data management and analysis

#### The primary outcomes

In the main evaluation, the two primary outcomes to be assessed from the baseline and endline survey will be:

1) The percentage of women delivering at the health facilities in the intervention and control areas. The difference of differences of the results from the intervention and control sites (obtained from baseline and end line surveys and from pre and post intervention facility data) will be computed to ascertain the influence of the intervention.

2) The percentage of poor women delivering in the health facilities in the intervention and control areas. Difference of difference analysis will be used to estimate the effect of the intervention among the poor.

The proportion of women delivering in the health facilities will be obtained from household surveys. Also, the total number of deliveries, and the types of deliveries (normal, assisted) will be obtained from the registers of the health facilities. Secondary outcomes will include the following:

• Birth outcomes [neonatal mortality rate (NMR),stillbirth rate]

• Proportion of women attending ANC 4, 3, 2 or once during the pregnancy

• Proportion of mothers who attended post natal care within 7 days

The difference in differences of the results from the intervention and control sites will be measured to ascertain the influence of the intervention. The equation that will be used to estimate the changes in health facility delivery and antenatal care attendance rates using the difference in differences analysis is given below.

E (Y*_i_*) =*β*_0_+*β*_1_Time+*β*_2_X_1_+*β*_3_Time*X_1_+*β*_4_CONFOUNDERS+*ε_i_*…… (1)

Y*_i_* = Value of i^th^ unit of Y_0_ = value of reference group at baseline

Time = Dummy variable (0=baseline; 1=follow-up)

X_1_ = Service and transport vouchers to clients

*β*_1_ = average effect of time over all groups

*β*_2_ = Difference in X_1_ intervention arm, compared to controls, at baseline

*β*_3_ = DD effect of interest: influence of X_1_ intervention over time on outcome

*β*_4_ = Average effects of confounding variables

CONFOUNDERS = vector of other potential confounding variables

*ε_i_* = errors for each unit at each time period, assumed to have normal (unknown) variance, mean of zero

The incremental costs for implementing a voucher system will be calculated and the incremental cost per additional delivery will be calculated as shown below:

Qualitative data will be transcribed and reconciled with the notes that were recorded during the interviews. Thematic and content analysis methods will be employed and qualitative methods software will be used during analysis of this qualitative data.

### Quality assurance

To ensure quality assurance the following measures are being taken:

• An implementation manual is being used to ensure that all activities are carried out correctly.

• All interviews are being done by well trained and senior researchers after orientation on the study purpose, objectives and methods.

• All the data collected during the implementation phase will be verified by supervisors review of voucher and health records.

• The instruments that are being used for data collection for the needs assessment, household survey and maternal audits were pre-tested before the survey is done.

• Key informant interviews are tape recorded in order to minimize loss of information.

• A study advisory group and the research partners in the FHS consortium provide technical advice and regular oversight of the implementation of the studies.

### Ethical considerations

Ethical approval to conduct the study was sought from the Institutional Review Board at the Makerere University School of Public Health and Uganda National Council for Science and Technology (UNCST) and an amendment was sought after the implementation of the pilot to cater to the changes made. Permission to conduct the study was obtained from the appropriate officers at the Ministry of Health and the districts where the study is being carried out. Written informed consent was obtained from all the study participants. At the conclusion of the project any information that could be used to link the respondents to the research data collected will be destroyed. Only individuals who have freely consented are being allowed to participate in the study, and no one will be coerced in any way to participate. The consent form used for the study details the aims, methods and anticipated benefits and risks of the research. It also informs the respondents that their identity and replies will be confidential, and that participating in the study will not result in any potential hazards. Lastly the participants are also informed that they have the right to abstain from the study, or to withdraw at any point.

## Results

### Baseline characteristics

Table 2 shows the baseline characteristics of the intervention district. The intervention district generally had a poor population and safe delivery coverage was only 34%. A total of 26 of the 39 health facilities in the district provide delivery care.

**Table 2:** Baseline characteristics of Kamuli and Pallisa Districts

Characteristic	Kamuli District	Pallisa District
Total population	680,500	480,000
Percent of births delivered in health facilities	34.0%	44.0%
Proportion below poverty line	35.9%	35.9%
Number and type of health facilities:		
Hospitals	2	2
Health Centre IV	3	2
Health Centre III	14	22
Health Centre II	51	13
Total	70	39

Sources: Ministry of Health (2008). Health Facilities Inventory. Uganda Ministry of Health, UNICEF country fact sheet

### Effects of the intervention

#### Changes in antenatal care utilisation

**Figure 1:**
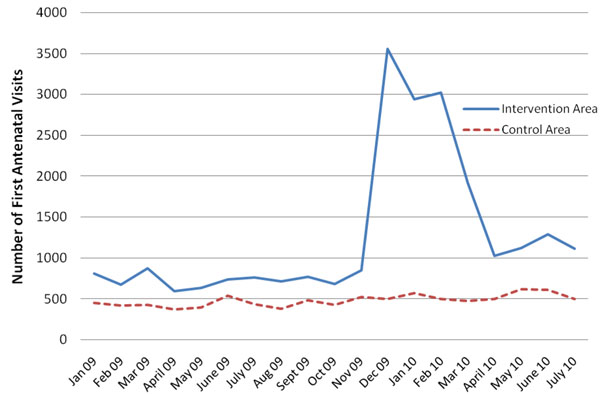
**Number of first antenatal care visits per month in the intervention and control areas**. Source: Health facility information system Pilot period begins December 2009; full implementation under revised scheme began in June 2010

Figure 1 shows the trend of first ANC visits in the intervention and control areas for Kamuli district for the period January 2009 to July 2010. There was a spike in the number of all ANC visits which coincided with the start of the pilot phase (December 2009), while the drop observed afterwards coincided with the end of the pilot phase (March 2010). When the pilot ended the project stopped distributing new vouchers, hence the mothers no longer had free transport for ANC services. The slight rise that was seen in June 2010 again coincided with the start of the implementation phase. During the implementation phase, vouchers were not offered for ANC services and that perhaps explains why the spike was smaller than that seen during the pilot. However ANC attendances especially the first visit was still much higher than what was observed in the previous years.

### Changes in health facility deliveries

**Figure 2:**
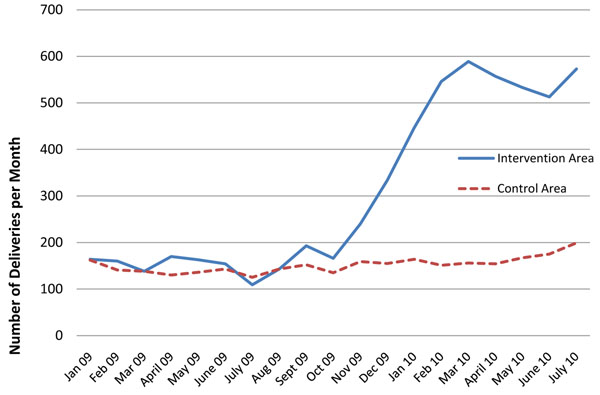
**Number of deliveries in health facilities per month in the intervention and control areas**. Source: Health facility information system Pilot period begins December 2009; full implementation under revised scheme began in June 2010

The response for health facility deliveries was much larger than expected, as the number of safe deliveries in the intervention area immediately jumped from <200 deliveries/month to over 500 deliveries/month in the intervention arm following the introduction of vouchers (Figure 2). Health facility deliveries began to rise in December 2009 when vouchers were introduced, and reached a peak in March 2010, and then started dropping. The drop coincided with the change in protocol (the voucher package changed from full transport coverage for all ANC, delivery and PNC package to delivery and PNC package) and start of the implementation phase.

### Changes in postnatal care attendances

**Figure 3:**
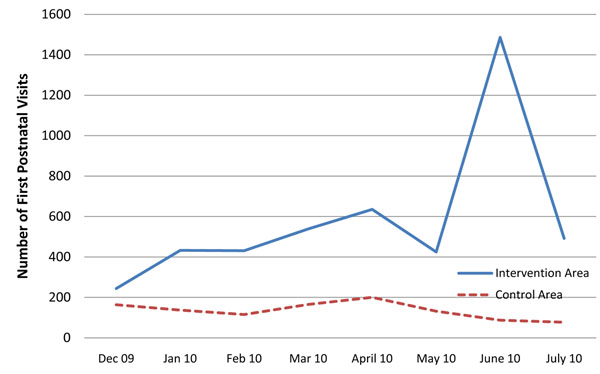
**Number of first postnatal care visits per month in the intervention and control areas**. Source: Health facility information system (postnatal record keeping began December 2009) Pilot period begins December 2009; full implementation under revised scheme began in Junel 2010.

Figure 3 shows the trend of first PNC visits to health facilities in the control and intervention arms for Kamuli District. Prior to the study, PNC visits were not systematically recorded in the data recorded at health facilities. The data therefore begins in December 2009, and shows a rise in PNC attendance as pilot and implementation phases of the project proceeded. The rise in PNC visits was not as sharp as the rise seen in the attendances of delivery and postnatal care.

## Discussion

The preliminary results from this study highlights how working differently in an innovative way with stakeholders led to the design of a project in which removal of barriers for obstetric care through provision of vouchers for transport and services dramatically led to an increase in access to health facility care for pregnant and newly delivered women. In the intervention areas, the response was much larger than expected, as the number of safe deliveries immediately jumped from <200 deliveries/month to over 500 deliveries/month. Such a rise in health facility utilisation for delivery care is unprecedented in Uganda, as the proportion of women delivering from health facilities has stagnated around 35-40% for over a decade. Since there is evidence to show that attendance of delivery by skilled health personnel reduces maternal mortality [24], this pilot study demonstrates that vouchers may be an effective way to rapidly increase institutional delivery in rural areas, and thereby help achieve MDG 5.

In addition to improvements in health facility deliveries, antenatal care, including those attending four or more visits, also increased. Attendance of four ANC visits is associated with improved maternal and newborn outcomes, and is recommended by the World Health Organisation [25-28]. However, in Uganda, although over 90% of women attend ANC at least once, fewer than half do so the recommended four times [1]. Thus, this study again proves that transport and service related factors are a key constraint to ANC attendance, but these can be reduced through special funding programs for the poor.

Use of vouchers in this pilot phase also led to dramatic increases in postnatal care attendance, hitherto an unavailable service. Attendance of postnatal care is a low cost intervention to improving maternal and newborn survival. In Uganda, it is estimated that only 12% of mothers attend PNC. Yet postnatal care has been found to be very effective in reducing both maternal and neonatal mortality [29].

This study also demonstrated that local transporters are an effective and yet untapped potential for transport and mobilisation of communities for improved maternal and newborn care. In this pilot, motorcyclists, motivated by a financial benefit accruing from transportation of pregnant and newly delivered mothers, actively mobilised women and transported them to health facilities. This was an unexpected finding, but helps to demonstrate the potential that is within communities [23].

Despite the increased utilisation of health facilities for ANC, delivery and PNC as a result of service and transport vouchers, the quality of care provided at health facilities could not be guaranteed. In addition, experiences through support supervision and in qualitative interviews revealed crowding at health facilities. Increased health facility utilisation without a corresponding increase in health workers may have resulted into reduced quality of some aspects of care. It is well known that increased utilisation without improved quality of care cannot lead to reduced maternal or neonatal mortality [30]. At the time the pilot was done there was a severe shortage in the number of health workers.

In this study, MakCHS worked with local stakeholders in efforts to improve health facility functionality through training, provision of drugs, equipment and supplies, and later on more health workers were recruited at the district. This close collaboration between MakCHS and the district to solve a local problem is an example of research-action and engagement approach to closing gaps [31]. These findings indicate that when scaling up interventions that can result in rapid increases in the utilization of services, provisions must be made to increase the capacity of the health system to offer services.

Interventions such as this one are useful for demonstrating how institutions of higher learning such as MakCHS can contribute to improving the capacity of health systems to deliver priority services to the poor. In order for this objective to be achieved, the intervention relied on a “learning and doing” approach. This approach allowed researchers from MakCHS to understand community and provider perspectives and influence practises in a manner that facilitates improvement of service delivery. For example, during the support supervision visits, they were able to contribute to the quality improvement efforts of the district. Secondly it allows the sharing of information between the implementers and researchers. This allowed the team to revise the intervention and to shape it in a manner that is suitable for the local context.

Another key concern for such small pilot projects are issues of scalability and sustainability. In the pilot phase of the current project, health providers and voucher scheme administrators were overwhelmed by the response in terms of increased utilisation for ANC, delivery care, and postnatal care. Although we are yet to perform a full cost-effectiveness analysis of such an intervention, the full package in the pilot phase was more than the project was able to finance from its resources, though estimated to cost less than 10 USD per delivery [23]. Consequently, the benefit package was scaled down to include only delivery and postnatal care for mothers and newborns. We recommend that if vouchers are to be adapted for maternal health, they should be tagged to outcomes which are high impact in terms of morbidity and mortality reduction and yet current coverage is low. These could include attending ANC four or more times, having a health facility delivery, access to referral for emergence obstetric and newborn care [29,30]. On the other hand, the vouchers could be managed as a community fund, with a community contribution, and a community decision of benefits. Such a strategy may be more acceptable to public officials, and could be more sustainable.

Community ownership of the project is also very important for sustainability. During the design and the implementation of this project, we worked closely with various stakeholders at national and district level to ensure that the design of the study is aligned to the government policies and practises, and also to ensure that key concerns are addressed at the design stage and during the implementation phase. We have also continued to share the results from the study with them. These interactions with stakeholders have allowed the team to generate the evidence that policy-makers request, which is essential for promoting the use of evidence to inform decisions related to country policies and programs.

Another challenge experienced in this pilot phase was in the management of vouchers. Challenges included delayed reimbursement and fraud. The payment of the vouchers for the health facilities entailed a two stage process, in which vouchers were first of all collected from the facilities, and then requisitions for payment processed and delivered. This was a long and tedious process. Two cases of fraud were reported. In one case the vouchers were stolen at the health facility, while in the second case fake vouchers were printed. These scenarios emphasise the need for the vouchers to have security features which will make it difficult to forge. It also indicates that at facility level security for the vouchers must be ensured. These cases of fraud came to our attention through members of the community, which reaffirms the need to involve the community in the voucher scheme.

This study demonstrates how ongoing reforms at Makerere University College of Health Sciences (MakCHS) are leading to new ways in which the College can work with local stakeholders to influence the health system with the ultimate aim of improving health status in Uganda. We think that this is a noble cause, given that universities like MakCHS are centres of excellence which could play an important role in societies such as in helping design evidence-based health interventions in low income countries. It also illustrates how interventions can be designed and implemented with the involvement of national and local stakeholders. This promotes the generation of evidence that is relevant in the local context enhancing the feasibility of the use of research findings for informing policy and programme processes within the country and other similar contexts [31].

Furthermore, the study highlights amendments and changes that were made to the protocol after the pilot study began, illustrating the benefits of doing a pilot study and of using evidence generated from monitoring activities to inform the designing and implementation of the intervention. Finally, when the study has been completed, the results will be able to demonstrate the degree to which using both demand and supply side initiatives can help improve maternal and newborn health in rural Uganda.

There are various limitations of the present study. First, the findings are preliminary, and occur after a very short period of only three months. The data used to report results in this paper were collected through the regular health information system, which is believed to suffer from inadequate supervision. However, we made efforts to improve data quality through training, sensitisation sessions, and supervision visits. In addition, we think that the community sensitisation and involvement helped to minimise fraud and moral hazard.

## Conclusions

This study has demonstrated that institutions of higher learning have the potential to work with stakeholders to use evidence to design interventions such as using the “learning-by-doing” approach that can enhance the ability of the health system to offer priority services that will result in the desired health outcomes. In this study, the use of transport and service vouchers were shown to be a potential strategy for increasing health facility utilisation for maternal and newborn services in poor rural areas, as it removes some key access barriers. In addition, motorcycle transporters are an untapped local resource for mobilising communities. Care must be taken to include measures that will ensure improved quality of care, which is required for reducing maternal and newborn mortality. It is also important to develop capacity for the proper management of the vouchers, and to ensure that other critical interventions such as family planning and immunization outreach services also receive high coverage. To be scalable, use of vouchers must be made with a high degree of community involvement. Finally, MakCHS, being a centre of excellence in research, needs to build capacity to provide implementation-related evidence for innovations that have been proved to work in other places, but need to be customised in Uganda.

## List of abbreviations used

MakCHS: Makerere University College of Health Sciences; MDG: Millennium Development Goal; ITN: insecticide treated nets; PNFP: private-not-for-profit; PFP: private-for-profit; HSD: health sub-district; PNC: postnatal care; ANC: antenatal care; HRS: household registration system; IPT: intermittent preventive treatment for malaria; NMR: neonatal mortality rate; UNCST: Uganda National Council for Science and Technology; FHS: Future Health Systems; DFID: UK Department for International Development; KI: key informant.

## Competing interests

The authors declare that they have no competing interests.

## Authors' contributions

GWP, EEK, PW, ER and OO initiated the concept for the study. EEK, PW and DHP co-wrote the manuscript. RH contributed to the formulation of the study, reviewed and provided substantial inputs into the protocol and came up with the analysis plan and the sample size calculation. FM and NK contributed towards the sample size calculation and the sampling procedure. JB, AM, GN, DS played key roles in the implementation and data collection phases. All authors read, provided substantial input and approved the final manuscript. EEK is the guarantor of the paper.
